# Port‐Site Seeding of Urothelial Carcinoma After Robotic‐Assisted Radical Prostatectomy Accompanied With Elevated Serum Levels of Carbohydrate Antigen 19‐9: A Case Report

**DOI:** 10.1155/criu/1618695

**Published:** 2026-04-11

**Authors:** Jun Kaneko, Sayuri Takahashi, Kazuyoshi Izukuri, Fusako Niimi, Mariko Tabata, Yuta Takeshima, Haruki Kume

**Affiliations:** ^1^ Department of Urology, The Institute of Medical Science, The University of Tokyo, Minato-ku, Tokyo, Japan, u-tokyo.ac.jp; ^2^ Division of Innovative Cancer Therapy, Advanced Research Center, Institute of Medical Science, The University of Tokyo, Minato-ku, Tokyo, Japan, u-tokyo.ac.jp; ^3^ Department of Urology, Faculty of Medicine, The University of Tokyo, Bunkyo-ku, Tokyo, Japan, u-tokyo.ac.jp

**Keywords:** CA19-9, case report, robotic surgery, urethelial carcinoma

## Abstract

Urothelial carcinoma (UC) of the bladder has a high recurrence rate. Non–muscle‐invasive bladder cancer (NMIBC) requires careful follow‐up after transurethral resection (TUR). Some cases of extravesical recurrence of UC have been reported following radical cystectomy or nephroureterectomy; however, few such cases following robot‐assisted radical prostatectomy (RARP) are known. A 77‐year‐old man presented to our hospital with high serum prostate‐specific antigen (PSA) levels and atypical urothelial cells. Prostate biopsy and photodynamic diagnosis (PDD)‐TURBT were performed. Pathological diagnoses were adenocarcinoma (Gleason score 3 + 4) of the prostate and UC (high grade, pT1 + Tis) of the bladder. Intravesical Bacillus Calmette–Guerin (BCG) was administered. One year after BCG therapy, examinations showed no evidence of UC recurrence; RARP was performed in another hospital on his will. Four months following RARP, elevated serum carbohydrate antigen 19‐9 (CA19‐9), palpable port‐site subcutaneous indurations, and intraperitoneal nodules were detected by blood test and positron emission tomography/computed tomography (PET/CT). The subcutaneous indurations were pathologically diagnosed as UC. Platinum‐based chemotherapy decreased the level of serum CA19‐9. Cancer progression with the elevation of serum CA19‐9 was observed during subsequent avelumab therapy, and enfortumab vedotin therapy was initiated. The patient died from cancer progression 30 months after extravesical recurrence of UC. RARP for patients with a past history of bladder tumor presents a risk of dissemination of UC due to urine leakage. Thorough examination for UC before RARP is recommended.

## 1. Introduction

Since the introduction of RARP, the number of prostatectomies has markedly increased. Many studies have shown that RARP is suitable and safe for the treatment of prostate cancer, and better preoperative, functional, and oncologic outcomes have been observed in patients receiving RARP [1]. Bladder cancer is the most common neoplasm of the urinary tract and the probabilities of recurrence and progression of NMIBC at 1 year after TURBT are estimated to range from 15% to 61% and from less than 1% to 17%, respectively [2]. A few cases of extravesical recurrence of UC have been reported after radical cystectomy or nephroureterectomy [3–5]; however, to our knowledge, there is only one report of peritoneal and port‐site seeding of bladder cancer subsequent to RARP [6].

Serum CA19‐9 is established as a tumor marker in cancers including pancreatic, gallbladder, and colon cancer. Several recent reports have suggested that a high CA19‐9 concentration is a poor prognostic factor in patients with UC [7].

We summarize the risks of port‐site seeding of UC after robotic surgery, and the relevance of serum CA19‐9 in UC recurrence, which may be useful information to surgeons in treating UC patients.

## 2. Case Presentation

**Figure 2:**
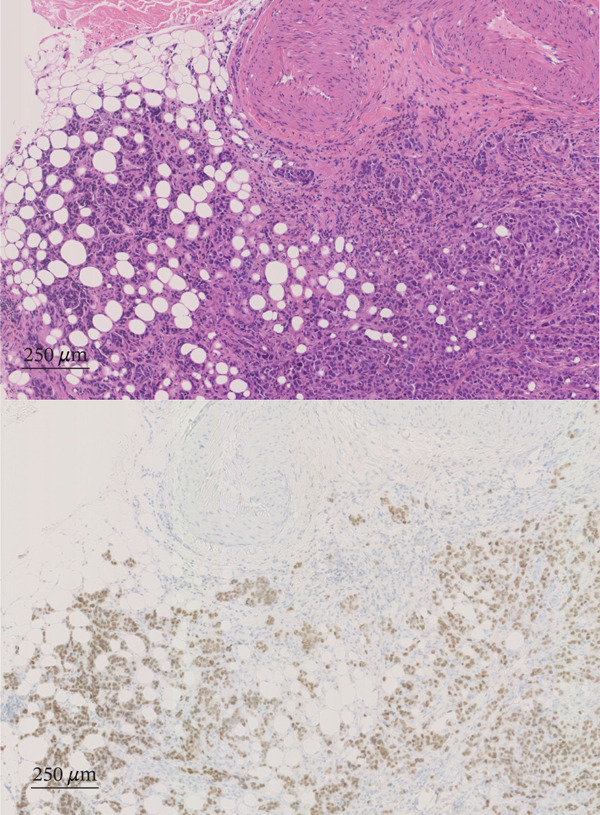
Pathological photos of subcutaneous induration. HE staining (upper) indicates urothelial carcinoma. GATA3‐positive cells were detected by immunostaining (lower).

A 77‐year‐old man presented to our department with an elevated serum PSA level of 8.29 ng/mL. Incidentally, microscopic hematuria and atypical urothelial cells were detected on urinalysis, and screening urine cytology was positive. A solid 2‐cm papillary bladder tumor was observed by cystoscopy. Contrast‐enhanced CT showed no metastasis. Prostate biopsy and PDD TURBT were performed. Pathological diagnoses were adenocarcinoma (Gleason score 3 + 4) of the prostate and urothelial carcinoma of the bladder (high grade, pT1 + Tis). Second TUR showed no evidence of malignancy. We performed intravesical BCG induction therapy for bladder cancer and initiated androgen deprivation therapy (ADT) because we were concerned that treatment for prostate cancer might be delayed. The patient declined further maintenance BCG therapy due to a concern for side effects. During BCG therapy and ADT, he underwent ureteral stenting for right hydronephrosis and hydroureter due to ureterovesical junction stenosis, which had seemingly arisen in the absence of recurrence or residual disease, as magnetic resonance urography, ureteroscopy, retrograde urography, and cytology of the right ureteral urine showed no evidence of malignancy. No recurrence of bladder tumor after the BCG therapy was confirmed by TUR‐biopsy. One year after BCG therapy, urine cytology was negative, and cystoscopy showed no recurrence of the tumor. Two months after the last ureteral stent exchange and cystoscopy, the patient visited another hospital and underwent RARP at the hospital without removing the ureteral stent, although we recommended long‐term surveillance from concern for dissemination of UC. The pathological diagnosis was acinar adenocarcinoma, Gleason score of 3 + 3, without evidence of extraprostatic extension or seminal vesical involvement. Surgical margins were negative (pathologic stage pT2aNxMx). Four months after RARP, the patient reported palpable subcutaneous indurations at the port sites, and the serum CA19‐9 was elevated to 674 U/mL. PET/CT showed increased uptakes in the intraperitoneal nodules and the port‐site indurations (Figure 1). An excisional subcutaneous biopsy was performed, and the indurations were pathologically diagnosed as recurrent UC (Figure 2). Chemotherapy by gemcitabine and carboplatin for 4 months decreased serum CA19‐9 level to 6.4 U/mL and reduced the metastatic lesions (Figure 3). However, multiple peritoneal dissemination recurrences were observed with elevated serum CA19‐9 levels after 5 months of avelumab maintenance therapy, and enfortumab vedotin was initiated while ureteral stents were changed regularly. The patient died from cancer progression after immune checkpoint inhibitors therapy for 2 years, and the serum PSA level was 0.01 ng/mL 1 month before death.

Figure 1Axial 18F‐FDG PET/CT fusion images before chemotherapy. Uptake in intraperitoneal nodules (a) and a port‐site subcutaneous induration (b) are demonstrated.

**Figure:**
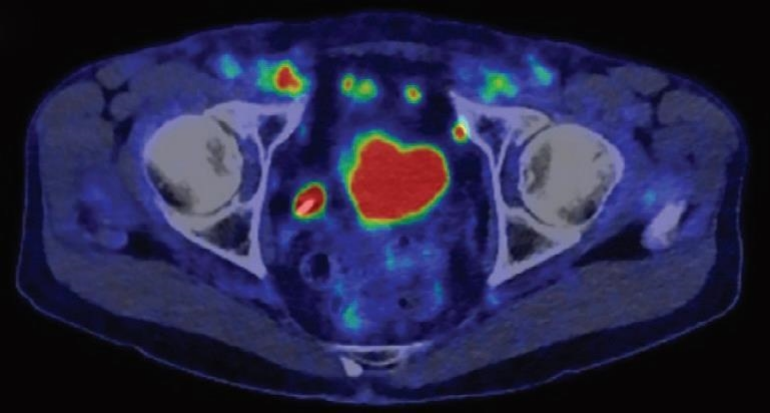
(a)

**Figure:**
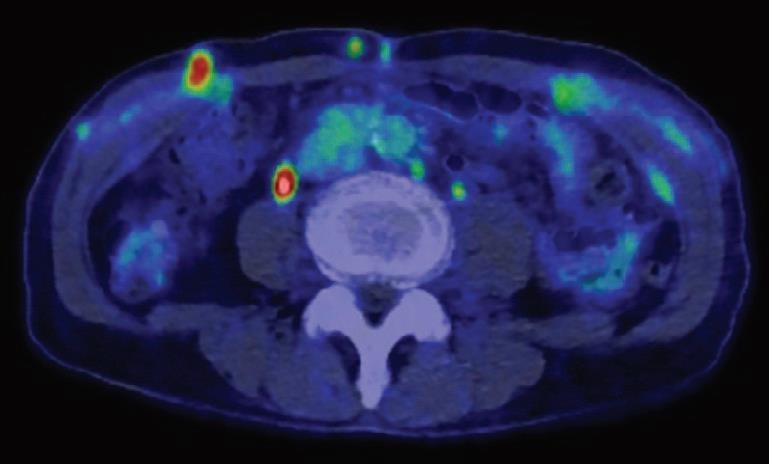
(b)

Figure 3CT images of intraperitoneal nodules (a, b) and a port‐site subcutaneous induration (c, d) before and after chemotherapy. Chemotherapy successfully reduced the lesions (a–b and c–d).

**Figure:**
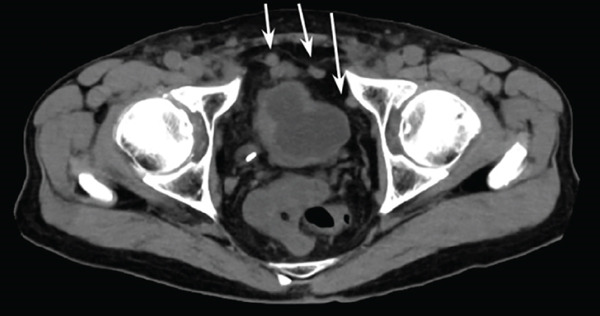
(a)

**Figure:**
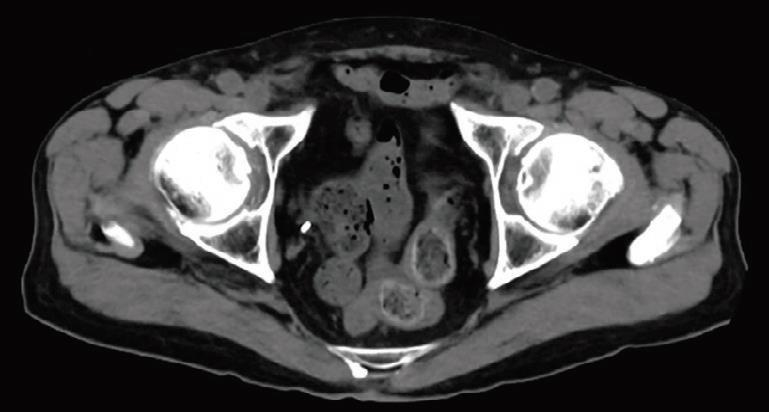
(b)

**Figure:**
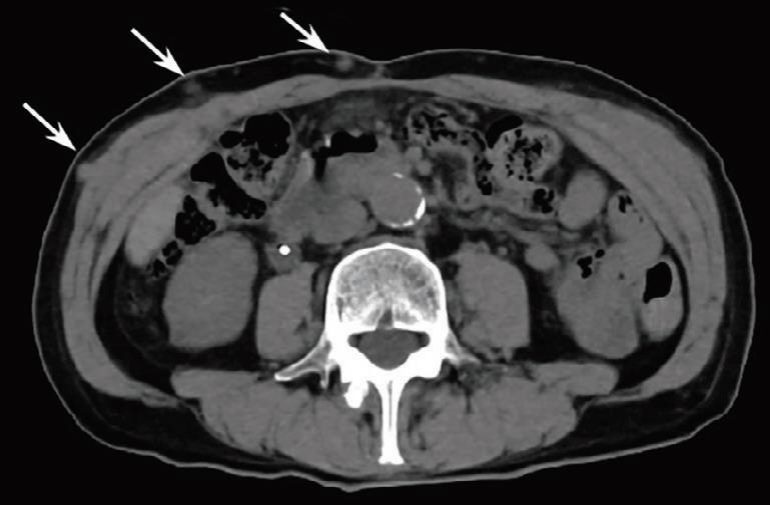
(c)

**Figure:**
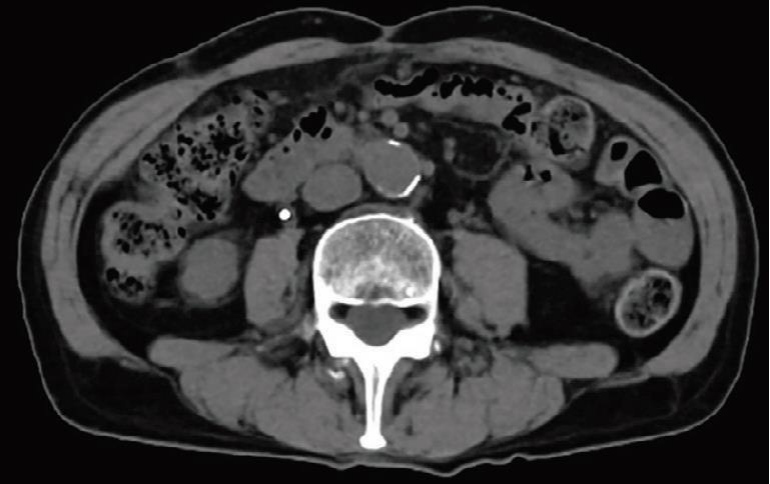
(d)

## 3. Discussion

Urothelial bladder cancer is one of the most common cancers by incidence in men and women [8], and it has a high rate of recurrence. T stage, grade, and the presence of carcinoma in situ (CIS) are known as the important prognostic factors. The probability of progression is estimated to be 29% at 1 year and 74% at 5 years in T1 G3 patients with CIS [2]. Intravesical BCG is widely used to reduce the risk of progression after transurethral resection in patients with NMIBC [9]. Five mechanisms of NIBC recurrence have been described: incomplete resection after TURBT, tumor reimplantation, growth of undetected tumors, new tumor formation, and a field change cancerization effect [10, 11]. To our knowledge, there is only one case report of peritoneal and port‐site seeding of UC after RARP [6]. In this report, although there were no metastatic lesions detected prior to RARP, cystoscopy was not conducted, which leaves open the possibility that an undiagnosed bladder tumor existed before RARP. In our case, extravesical recurrence occurred in less than 6 months after RARP, although intravesical BCG was administrated and there was no intravesical recurrence. We presume that undetected tumor reimplantation might be iatrogenically caused by the leakage of urine and high intra‐abdominal air pressure during the robotic surgery, and extravesical recurrence and port‐site subcutaneous indurations occurred as a result. In addition, the presence of the right ureteral stent might have interfered with bladder neck dissection or vesicourethral anastomosis during surgery, causing the risk of extravesical recurrence.

Interestingly, in our case, rapid elevation of serum CA19‐9 occurred in parallel with recurrence of UC. Serum CA19‐9 is known as a tumor marker for pancreatic and gastrointestinal cancer, but sometimes benign diseases such as hydronephrosis are also the cause of elevated serum CA19‐9 [12]. In addition, serum CA19‐9 sometimes rises in bladder cancer and it is associated with worse oncological outcome in patients with invasive bladder cancer [13, 14]. Hydronephrosis of this patient was well controlled by the ureteral stent during the chemotherapy; therefore, the factor of serum CA19‐9 elevation seemed to occur from the extravesical and port‐site seeding of UC. Khan MF et al. stated that serum CA19‐9 was markedly reduced by the induction of cancer chemotherapy [7]. Our case also showed reduction of serum CA19‐9 after chemotherapy. Thus, serum CA19‐9 may be a useful marker that reflects the progression of UC metastasis and recurrence.

Robotic surgery is becoming an increasingly popular modality due to its being a less invasive and more advanced technique. However, urine leakage during RARP may present a risk of dissemination of cancer cells. Cystoscopy, TUR‐biopsy, or urine cytology on patients who were diagnosed with UC in the past are recommended before RARP.

## Author Contributions

Jun Kaneko and Sayuri Takahashi drafted the manuscript. Haruki Kume supervised. Kazuyoshi Izukuri, Fusako Niimi, Mariko Tabata, and Yuta Takeshima assisted in perioperative care.

## Funding

No funding was received for this manuscript.

## Disclosure

All authors reviewed and approved the final manuscript.

## Ethics Statement

Ethical approval was not required for this case report.

## Consent

Written informed consent was obtained from the patient for publication of this case report.

## Conflicts of Interest

The authors declare no conflicts of interest.

## Data Availability

Data sharing is not applicable to this article as no datasets were generated or analyzed during the current study.
